# Identification of Vital and Dispensable Sulfur Utilization Factors in the *Plasmodium* Apicoplast

**DOI:** 10.1371/journal.pone.0089718

**Published:** 2014-02-21

**Authors:** Joana M. Haussig, Kai Matuschewski, Taco W. A. Kooij

**Affiliations:** 1 Parasitology Unit, Max Planck Institute for Infection Biology, Berlin, Germany; 2 Institute of Biology, Humboldt University, Berlin, Germany; University of Cambridge, United Kingdom

## Abstract

Iron-sulfur [Fe-S] clusters are ubiquitous and critical cofactors in diverse biochemical processes. They are assembled by distinct [Fe-S] cluster biosynthesis pathways, typically in organelles of endosymbiotic origin. Apicomplexan parasites, including *Plasmodium*, the causative agent of malaria, harbor two separate [Fe-S] cluster biosynthesis pathways in the their mitochondrion and apicoplast. In this study, we systematically targeted the five nuclear-encoded sulfur utilization factors (SUF) of the apicoplast [Fe-S] cluster biosynthesis pathway by experimental genetics in the murine malaria model parasite *Plasmodium berghei*. We show that four *SUFs,* namely *SUFC*, *D*, *E*, and *S* are refractory to targeted gene deletion, validating them as potential targets for antimalarial drug development. We achieved targeted deletion of *SUFA*, which encodes a potential [Fe-S] transfer protein, indicative of a dispensable role during asexual blood stage growth *in vivo*. Furthermore, no abnormalities were observed during *Plasmodium* life cycle progression in the insect and mammalian hosts. Fusion of a fluorescent tag to the endogenous *P. berghei SUFs* demonstrated that all loci were accessible to genetic modification and that all five tagged SUFs localize to the apicoplast. Together, our experimental genetics analysis identifies the key components of the SUF [Fe-S] cluster biosynthesis pathway in the apicoplast of a malarial parasite and shows that absence of *SUFC*, *D*, *E*, or *S* is incompatible with *Plasmodium* blood infection *in vivo*.

## Introduction

Iron-sulfur [Fe-S] clusters are small inorganic cofactors that are present in most organisms. Proteins containing [Fe-S] clusters are involved in numerous biological processes, ranging from mitochondrial oxidative phosphorylation [1] and photosynthesis [2] to DNA replication [3], DNA repair [4], ribosome biogenesis [5], and regulation of gene expression [6]. Accordingly, the list of [Fe-S] cluster-containing proteins is continuously expanding. Although early *in vitro* studies suggested a spontaneous assembly [7], [Fe-S] clusters are not formed spontaneously in living cells but rather assembled through distinct [Fe-S] biosynthesis pathways.

Bacteria harbor the sulfur utilization factor (SUF) and iron-sulfur cluster (ISC) systems for assembly of [Fe-S] clusters. In *Escherichia coli*, the ISC system is thought to mediate housekeeping functions, whereas the SUF system was shown to be especially important under stress conditions such as iron starvation [8], [9]. However, deletion of individual operons is not lethal [10], [11].

In eukaryotes, [Fe-S] biogenesis machineries are thought to have evolved from their bacterial counterparts that had been acquired by endosymbiosis [12]. Different [Fe-S] cluster assembly systems are required for biogenesis in distinct cellular compartments, namely the ISC system in the mitochondrion and the SUF system in plastids. The mitochondrial ISC proteins were found to supply [Fe-S] clusters to the mitochondrial [Fe-S] proteins and to [Fe-S] proteins in the cytosol [13], where the cytosolic iron-sulfur protein assembly (CIA) machinery is responsible for the maturation of cytosolic [Fe-S] proteins [14].

In spite of the differences between bacteria and eukaryotes, the basic principles of [Fe-S] clusters biogenesis seem to be conserved. First, the [Fe-S] cluster is assembled *de novo* onto a scaffold protein. For this step, sulfur is mobilized from cysteine by a cysteine desulfurase (SufS, IscS, NifS) [15]. The iron source is mostly unknown. Second, the [Fe-S] cluster is transferred from the scaffold protein to a target apoprotein and assembled into the polypeptide chain. The most common [Fe-S] clusters are rhombic [2Fe-2S] or cubic [4Fe-4S]. More complex structures have also been described, some of which include additional heavy metals [16].

The SUF system in *E.coli*, for instance, consists of six genes organized in the *sufABCDSE* operon [11]. SufS acts as cysteine desulfurase that provides the sulfur for the [Fe-S] cluster and SufE has been shown to interact with SufS to enhance its activity up to 50-fold [17], [18]. SufB, C, and D form a functional complex serving as a [Fe-S] scaffold [19], [20] and also enhancing SufS function [18]. SufC was shown to contain ATPase activity in *Erwinia chrysanthemi* [21] and the crystal structure of *E. coli* SufC confirmed it to be an ABC-type ATPase [22]. SufA interacts with the SufBCD complex to accept [Fe-S] clusters formed *de novo* [19].

In *Plasmodium*, a genus of eukaryotic, single cell parasites that are the causative agents of malaria, components of the SUF, ISC, and CIA system have been identified by bioinformatic analyses [23]–[25]. Like in other eukaryotes, the ISC system is predicted to localize to the mitochondrion and the SUF system to the apicoplast, a vestigial, non-photosynthetic plastid of red algal origin [24]. In *Plasmodium*, all components of the SUF system are nuclear-encoded, except for SUFB, which is encoded in the small circular apicoplast genome [26].

A biochemical study focusing on the *P. falciparum* plastid SUF system provided evidence that SUFC localizes to the apicoplast [27]. In bacteria and plants, SUFC interacts with SUFB [21], [28], [29], which was confirmed for the corresponding *P. falciparum* proteins [27]. In *Arabidopsis thaliana*, SUFB and SUFC both display ATPase activity [29], whereas the bacterial SUFB seems to lack this activity. ATPase activity of recombinant *Pf*SUFB and *Pf*SUFC proteins provided supporting evidence for the evolutionary conservation of the plastid SUF system between plants and apicomplexan parasites [27]. *Pf*SUFE and *Pf*SUFS have also been assigned to the apicoplast, while *Pf*SUFS was shown to functionally complement *E. coli* deficient in *SufS* [30].

Data-mining and bioinformatic analysis of potential *Plasmodium* [Fe-S] cluster-containing proteins revealed at least 31 candidates, seven of which are predicted to localize to the apicoplast (Table 1). These proteins are involved in diverse pathways, such as mevalonate-independent isoprenoid biosynthesis, lipoic acid metabolism, and biogenesis of [Fe-S] clusters itself. Because some proteins are components of essential biosynthesis pathways, most notably the DOXP pathway of isoprenoid biosynthesis [31], plastid [Fe-S] cluster assembly is likely essential for parasite survival.

**Table 1:** Confirmed and predicted [Fe-S] cluster-containing proteins in *Plasmodium*.

*P. berghei* a	*P. falciparum* a	Annotation	PlasmoAP^b^	ApicoAP^b^	PlasMit^b^	MitoProtII^b^
**APICOPLAST** a						
PBANKA_020870	PF3D7_0104400	IspH/LytB, 4-hydroxy-3-methylbut-2-enyl diphosphate reductase	−/−	no SP	possibly	0.5404
PBANKA_050700	PF3D7_1022800	IspG/GcpE, 4-hydroxy-3-methylbut-2-en-1-yl diphosphate synthase	+/++	ATP	non-mito	0.8285
PBANKA_070700	PF3D7_0823600	LipB, lipoate-protein ligase	+/++	no SP	non-mito	0.9748
PBANKA_081190	PF3D7_0910800	nucleotide binding protein, putative	++/++	ATP	non-mito	0.9760
PBANKA_112110	PF3D7_0622200	radical SAM protein, putative	+/++	ATP	non-mito	0.9952
PBANKA_135750	PF3D7_1344600	LipA, lipoyl synthase	++/++	ATP	non-mito	0.6838
PBANKA_141660	PF3D7_1318100	ferredoxin, putative	++/++	ATP	non-mito	0.8117
**MITOCHONDRION** a						
PBANKA_061790	PF3D7_0720400	ferrodoxin reductase-like protein	−/++	no SP	possibly	0.8670
PBANKA_082810	PF3D7_0927300	fumarat hydratase, putative	−/++	no SP	non-mito	0.8939
PBANKA_090930	PF3D7_1139700	adrenodoxin reductase, putative	−/+	no SP	possibly	0.0610
PBANKA_122950	PF3D7_0614800	endonuclease III homologue, putative	−/++	no SP	non-mito	0.5453
PBANKA_130330	PF3D7_1439400	ubiquinol-cytochrome c reductase iron-sulfur subunit, putative	−/++	no SP	possibly	0.9782
PBANKA_135520	PF3D7_1342100	aconitate hydratase	−/++	no SP	possibly	0.8460
PBANKA_142880	PF3D7_1212800	iron-sulfur subunit of succinate dehydrogenase	−/++	no SP	possibly	0.1521
PBANKA_143040	PF3D7_1214600	adrenodoxin-type ferredoxin, putative	−/++	no SP	possibly	0.8299
**NUCLEUS or CYTOPLASM** a						
PBANKA_011240	PF3D7_0614200	conserved Plasmodium protein, unknown function	−/−	no SP	non-mito	0.0141
PBANKA_083490	PF3D7_0934100	XPD/ERCC2, DNA excision-repair helicase, putative	−/−	no SP	non-mito	0.0106
PBANKA_091970	PF3D7_1128500	conserved protein, unknown function	−/−		non-mito	0.1301
PBANKA_101520	PF3D7_1429500	diphthamide synthesis protein, putative	−/−	no SP	non-mito	0.2517
PBANKA_102890	PF3D7_1413800	diphthamide synthesis protein, putative	−/−	no SP	possibly	0.0176
PBANKA_103410	PF3D7_1408400	DNA-repair helicase, putative	−/−	no SP	possibly	0.0139
PBANKA_103530	PF3D7_1406900	radical SAM protein, putative	−/+	no SP	possibly	0.1722
PBANKA_133970	PF3D7_1324500	DEAD box helicase, putative	−/−	no SP	non-mito	0.4691
**SUBCELLULAR LOCALIZATION NOT PREDICTED** a						
PBANKA_011230	PF3D7_0614100	conserved Plasmodium protein, unknown function	−/++	no SP	possibly	0.2399
PBANKA_070600	PF3D7_0824600	anamorsin related protein, putative	−/−	no SP	non-mito	0.1138
PBANKA_081200	PF3D7_0910900	DNA primase large subunit, putative	−/++	no SP	possibly	0.0509
PBANKA_090570	PF3D7_1143300	DNA-directed RNA polymerase I, putative	−/−	no SP	non-mito	0.0677
PBANKA_100950	PF3D7_1435300	NAD(P)H-dependent glutamate synthase, putative	−/−	no SP	non-mito	0.0131
PBANKA_114410	PF3D7_1368200	RNAse L inhibitor protein, putative	−/−	no SP	possibly	0.0535
PBANKA_123970	PF3D7_0524900	tRNA-YW synthesizing protein, putative	−/0	no SP	non-mito	0.0527
PBANKA_144250	PF3D7_1227800	histone S-adenosyl methyltransferase, putative	−/−	no SP	non-mito	0.0660

a Gene IDs of the *P. berghei* and *P. falciparum* orthologs and the predicted localizations of the proteins were retrieved from PlasmoDB (http://PlasmoDB.org) or identified by similarity searches using *A. thaliana* [Fe-S] cluster proteins as query sequences [41].

b Putative targeting of the *P. falciparum* [Fe-S] cluster-containing proteins to the apicoplast or mitochondrion was predicted using four different algorithms. PlasmoAP [51] indicates the likelihood of the presence of the required signal peptide followed by the likelihood of an apicoplast localization (“−” = unlikely, “0″ = undecided, “+” = likely, “++” = very likely). ApicoAP [52] is a different algorithm that can identify apicoplast proteins in multiple *Apicomplexa* (“No SP” = no signal peptide, “No ATP” = signal peptide but no transit peptide, “ATP” = apicoplast targeted protein). PlasMit [53] predicts the likelihood of a mitochondrial localization for *P. falciparum* proteins (“non-mito” (99%), “possibly” (91%), and “mito” (99%)). MitoProtII [54] gives a probability score for the likelihood of mitochondrial localization but is not optimized for *Plasmodium* sequences.

We previously reported that a *Plasmodium*-specific component of the apicoplast [Fe-S] cluster biosynthesis pathway, nitrogen fixation factor U (NifU)-like domain containing protein (NFUapi), can be deleted in the murine malaria model parasite *P. berghei* and plays an auxiliary role in liver stage merozoite formation [32]. Here, we present a systematic experimental genetics analysis of the *P. berghei* apicoplast SUF system. We show that four of five nuclear-encoded *P. berghei SUF* genes are refractory to gene deletion and, hence, can be considered likely essential for blood stage proliferation. Endogenous tagging confirmed accessibility of the loci to targeted gene modification and the resulting fluorescent fusion proteins showed co-localizations with an apicoplast resident protein.

## Results

### The Principal Components of the *Plasmodium berghei SUF* Pathway are Refractory to Targeted Deletion *in vivo*

**Figure 1:**
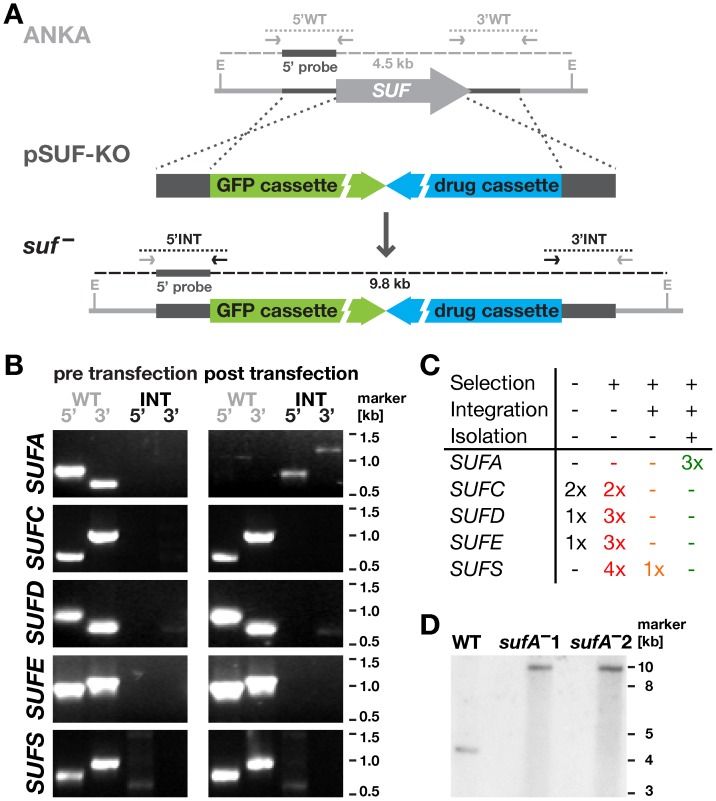
Systematic gene targeting of *Plasmodium berghei SUF* genes. (A) Replacement strategy to delete the five nuclear-encoded *PbSUF* genes. The respective ANKA strain wild type (WT) *SUF* loci were targeted with replacement plasmids (pKO) containing upstream 5′ and downstream 3′ regions (dark gray bars) flanking the open reading frames (light gray arrow), a high-expressing GFP cassette (green), and the *hDHFR-yFcu* drug-selectable cassette (blue). Integration-specific (5′INT and 3′INT) and wild type-specific (5′WT and 3′WT) primer combinations (Table S1) are indicated by arrows; expected PCR fragments by dotted lines. The probe used for Southern blot analysis of the two isogenic *sufA* ^–^ parasites lines corresponds to the 5′ integration sequence and hybridized to EcoRI (E) restriction-digested gDNA; expected fragments and their sizes are indicated by gray dashed lines. (B) Representative diagnostic PCR results of the *SUF* loci of WT ANKA (pre transfection) and drug-selected (post transfection) parasites are shown. For *SUFA*, diagnostic PCR of isogenic gene deletion parasites confirms successful integration and absence of WT parasite contamination. (C) Overview of all transfection experiments summarizing the number of times no pyrimethamine-resistant parasites were selected (black), *selection* of pyrimethamine-resistant parasites was achieved (red), *integration*-specific PCR demonstrated targeted deletion of the *SUF* gene (orange), and *isolation* of WT-free, isogenic recombinant parasites (green). (D) Southern blot analysis of two isogenic *sufA* ^–^ parasite lines reveals the expected size shifts.

We first wanted to investigate whether the *SUF* genes of the *Plasmodium* apicoplast [Fe-S] cluster biosynthesis pathway were susceptible or refractory to targeted gene deletion. For this systematic genetic characterization, we employed the murine malaria model system *P. berghei* that permits *in vivo* selection of recombinant parasites. We attempted to generate loss-of-function mutants of the five nuclear-encoded components (Fig. 1). *SUFB* (PBANKA_API0012) is encoded on the apicoplast circular genome and cannot be targeted by the available transfection technologies.

We employed currently available experimental genetics techniques [33], [34] to delete the open reading frames of all five target genes (Fig. 1A). Upon successful double homologous/ends out recombination events, recombinant parasites are predicted to contain high-expressing GFP- and drug-selectable cassettes in place of the respective genes. After positive selection with the antimalarial drug pyrimethamine, potential recombinant parasites were isolated by flow cytometry [35] and subjected to genotyping by diagnostic PCR (Fig. 1B and Table S1).

In three independent transfection attempts, we were able to select *sufA* ^–^ parasites (Fig. 1B and C). In marked contrast, we were unable to generate recombinant gene deletion parasites for *SUFC* (PBANKA_102920), *SUFD* (PBANKA_094350), *SUFE* (PBANKA_030380), or *SUFS* (PBANKA_061430) in four independent transfection experiments. In a single transfection experiment targeting *SUFS*, we observed integration-positive PCR products. Repeated attempts to isolate gene deletion mutants via flow cytometry or limiting dilution cloning always resulted in a wild type (WT)-containing population, as tested by a WT-specific PCR reaction that only amplifies the WT locus, suggesting a requirement for the presence of *SUFS* as shown previously for other essential genes, *e.g.* the chloroquine resistance transporter [36]. Refractoriness to targeted gene deletion strongly suggests that the four *SUF* genes are crucial for propagating a successful blood infection, the phase of the *Plasmodium* life cycle where transfection is performed.

Collectively, our reverse genetics findings indicate that four out of five *Plasmodium* SUF proteins, namely SUFC, SUFD, SUFE, and SUFS, perform critical functions during blood infection of the malarial parasite.

### *SUFA* is Dispensable for *Plasmodium* life Cycle Progression

Repeated successful deletion of *SUFA* (PBANKA_123740) during our transfection experiments already indicated non-vital roles of the target gene for erythrocytic parasite propagation. In order to determine potential *in vivo* roles during the parasite life cycle, we selected two isogenic *sufA* ^–^ populations. Genotyping, including Southern blot analysis (Fig. 1D), confirmed the homogenous presence of *sufA* ^–^ parasites only.

**Figure 2:**
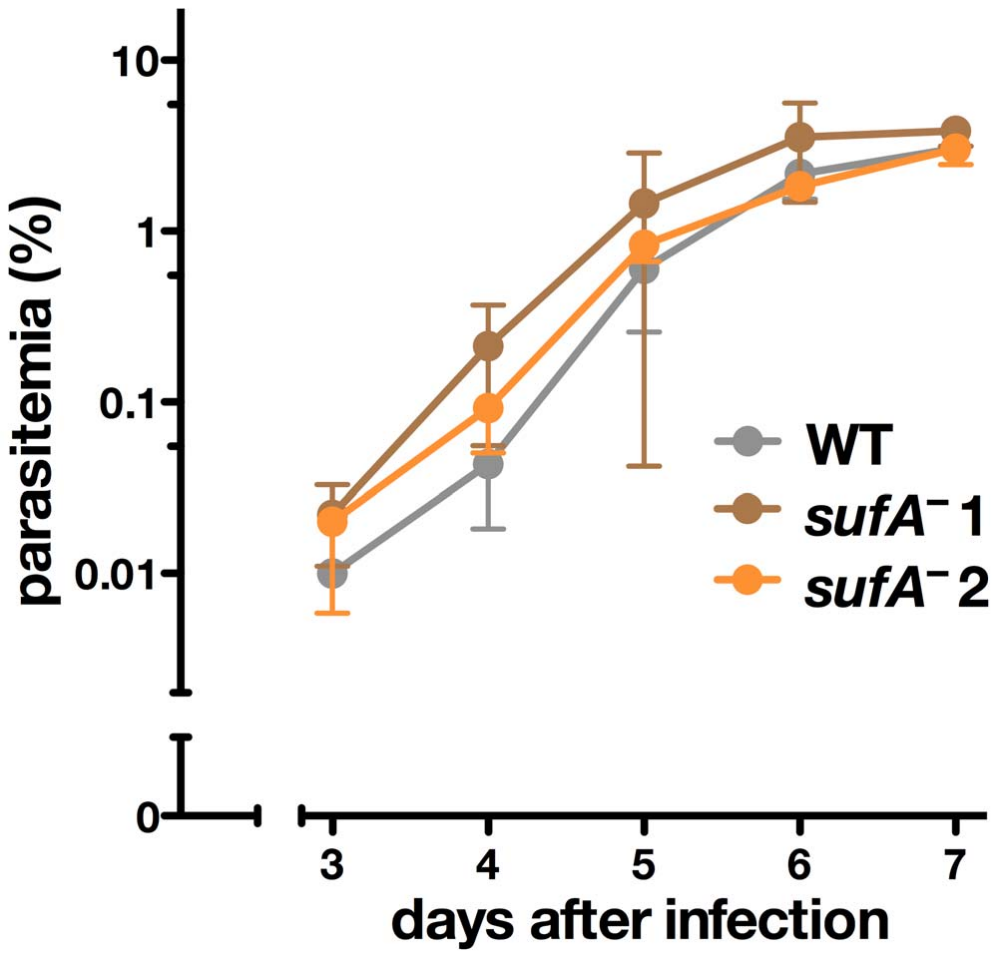
*SUFA* is dispensable for blood infection *in vivo*. Parasitemias of *sufA* ^–^-infected animals in comparison to mice infected with wild type (WT) parasites. Female C57BL/6 mice (WT, *n* = 3; *sufA* ^–^1 and 2, *n* = 5 each) were injected intravenously with 10,000 freshly dissected salivary gland sporozoites and infection was monitored by microscopic examination of Giemsa-stained blood films. The two isogenic *sufA* ^–^ parasite lines (brown and orange lines) and WT parasites (gray line) showed equal pre-patent periods (three days) and similar exponential parasite growth (*P*>0.05; two-way ANOVA followed by Bonferroni posttests).

To mimic a natural infection, we propagated the two selected *sufA* ^–^ populations through the mosquito vector, female *Anopheles stephensi*, and isolated sporozoites from infected salivary glands. Intravenous injection of 10,000 wild type (WT) or *sufA* ^–^ sporozoites were performed to infect and monitor transmission to C57BL/6 mice (Fig. 2). This analysis showed the typical pre-patent period, which is the time until first detection of blood stage parasites in peripheral blood following infection with sporozoites and which includes the liver stage development, of three days in all animals tested. Moreover, during the following days, *sufA* ^–^ and WT-infected animals displayed similar development of parasitemias. All parasites first replicated exponentially before entering a plateau phase, once the parasitemia was close to 1%. The exposure of three naïve C57BL/6 mice to bites of five infected mosquitoes also resulted in successful natural transmission in two mice. In conclusion, this analysis demonstrated that *SUFA* does not play important roles in establishment and propagation of an erythrocytic infection and, hence, is not a valid target for rational drug design.

**Figure 3:**
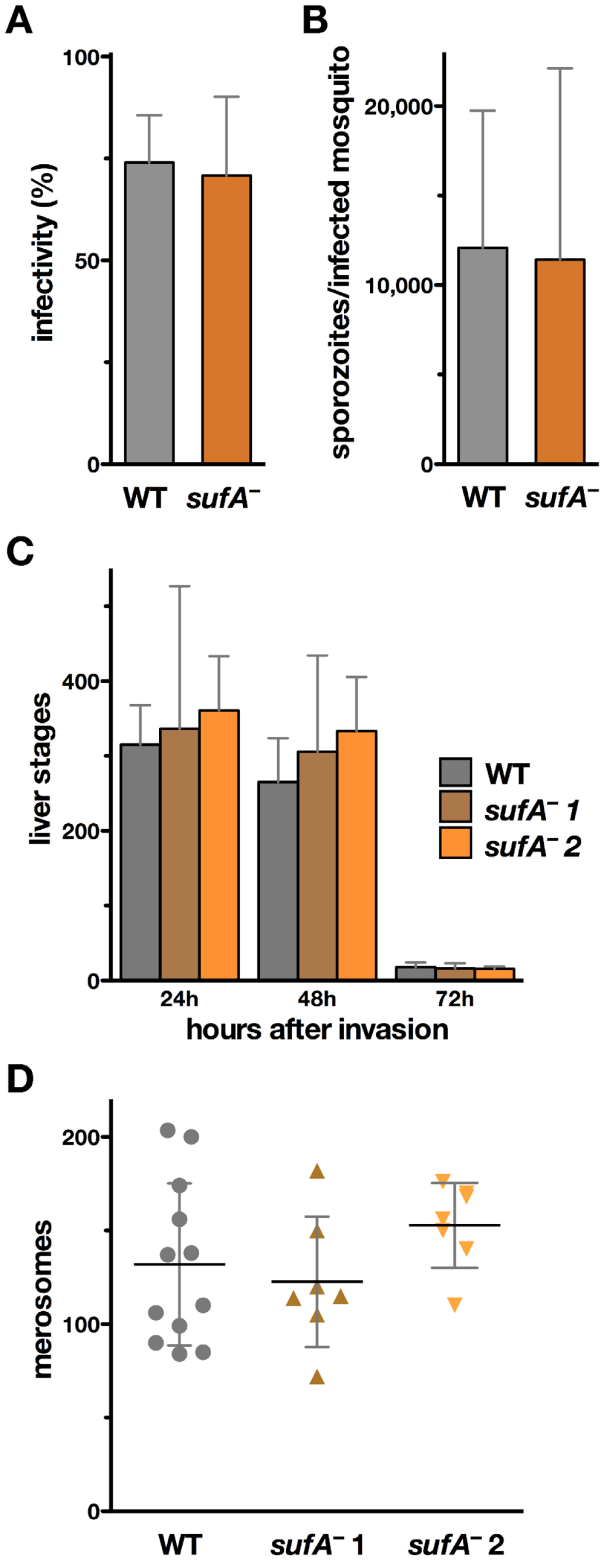
Normal development of *sufA* ^–^ parasites in the mosquito vector and during liver cell infection. (A) Percentage of *A. stephensi* mosquitoes infected with WT (gray, *n* = 4) and *sufA* ^–^ (brown, *n* = 6) parasites. Shown is the mean percentage (± S.D.) from independent mosquito feeding experiments. For *sufA* ^–^ infections, both isogenic strains, *sufA* ^–^1 (*n* = 4) and *sufA* ^–^2 (*n* = 2), were transmitted to mosquitoes and data combined. (B) Mean sporozoite number (± S.D.) in salivary glands (days 17–21 after infection) from the same independent mosquito feedings as shown in panel A (WT, *n* = 4; pooled *sufA* ^–^, *n* = 6). (C) Liver stages development of WT and *sufA* ^–^ parasites in cultured hepatoma cells. Shown are mean numbers (± S.D.) of intracellular parasites at the time points indicated from two experiments done in quadruplicate each. (D) Merosome formation at 72 h after infection. Shown are mean values (± S.D.). None of the *sufA* ^–^ data points were significantly different from the corresponding WT values (*P*>0.05; non-parametric, two-tailed Mann-Whitney’s test).

Successful *sufA* ^–^ sporozoite isolation and infection of mice were already indicative of unaltered life cycle progression in the absence of *SUFA*. To exclude modest defects, we systematically assessed parasite development in the mosquito vector and during pre-erythrocytic growth (Fig. 3). Transmission to mosquitoes and sporogony were indistinguishable from WT parasites, as exemplified by similar infectivity to mosquitoes (Fig. 3A) and normal sporozoite numbers in salivary glands, the final target organ in the mosquito vector (Fig. 3B). Also when we quantified intrahepatic parasite stages in cultured hepatoma cells (Fig. 3C) and merosomes from culture supernatants, representing emerging merozoites (Fig. 3D), we could not distinguish *sufA^–^* from WT parasites. In both parasite lines, merosomes are released into the supernatant between 48 and 72 h after infection, leading to a remarkable drop in parasite numbers in hepatoma cells seen at 72 h after infection.

Together, our analysis demonstrates that absence of *SUFA* is compatible with effective host switch, parasite stage conversion, and population expansion throughout the entire *Plasmodium* life cycle.

### Apicoplast Localization of *Plasmodium* SUF Proteins

**Figure 4:**
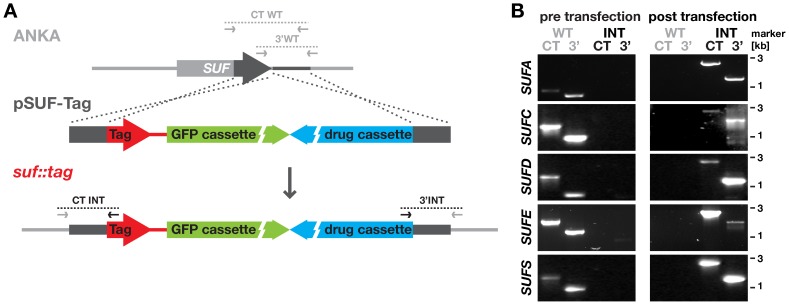
Control transfections of *Plasmodium berghei SUF* genes and generation of fluorescently tagged SUF parasite lines. (A) Replacement strategy to generate stable parasite lines that express the endogenous SUF proteins fused to an mCherry-3xMyc tag (red). The respective ANKA strain wild type (WT) *SUF* loci were targeted with replacement plasmids (pSUF-Tag) containing carboxy terminal (CT) and downstream 3′ regions (dark gray bars), a high-expressing GFP cassette (green), and the *hDHFR-yFcu* drug-selectable cassette (blue). Integration-specific (CT INT and 3′INT) and wild type-specific (CT WT and 3′WT) primer combinations (Table S1) and expected fragments are indicated by arrows and dotted lines, respectively. (B) PCR-based genotyping of the *suf::tag* parasites to verify successful fusion of the respective *SUF* genes with the mCherry-3xMyc tag and WT-free isolation of the recombinant *suf::tag* parasites. Note that the 3′ WT- and integration-specific PCRs are identical to those designed for targeted gene deletion (Figure 1).

In order to gain independent confirmation that the four essential *SUF* loci are susceptible to genetic manipulation, we targeted *SUFC*, *D*, *E*, and *S* along with *SUFA* by double homologous/ends-out recombination introducing a carboxy-terminal in-frame fusion with the combined mCherry-3xMyc tag (Fig. 4A), using the same strategy we previously employed to tag NFUapi [32]. Upon successful recombination, selection of recombinant parasites by the antifolate pyrimethamine, and WT-free isolation by flow cytometry, we readily obtained all five desired recombinant parasites, *i.e. sufA::tag*, *sufC::tag*, *sufD::tag*, *sufE::tag*, and *sufS::tag*, after the first transfection attempt (Fig. 4B). In addition to demonstrating accessibility of the loci to genetic modification, the parasite populations provided templates for the verification of the respective diagnostic PCRs of the 3′ integration of the unsuccessful gene deletion attempts (Fig. 1B), which use the same homologous sequence for integration.

**Figure 5:**
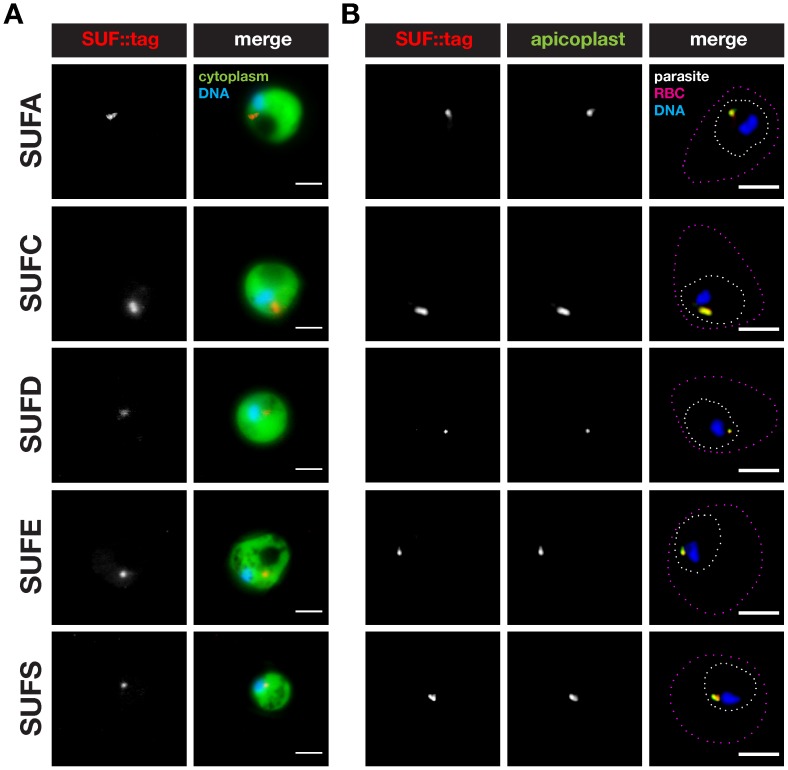
Localization of *Plasmodium berghei* SUFs to the apicoplast in blood stages. (A) Epifluorescent micrographs of live *suf::tag* parasite-infected mouse erythrocytes. Shown are representative micrographs of trophozoite stage parasites. The parasite cytoplasm is labeled by GFP and parasite nuclei by the DNA-dye Hoechst. Bars, 2 µm. (B) Co-staining of fixed trophozoite stage *suf::tag* parasites using anti-mCherry antibodies and anti-sera against acyl carrier protein (ACP), a signature protein of the apicoplast. Substantial overlap can be observed between the SUF::tag proteins and the signature apicoplast protein in a small structure, *i.e.* the apicoplast. The outlines of the parasite and the infected red blood cell (RBC) are indicated by white and magenta dotted lines, respectively. Nuclei were stained with the DNA-dye Hoechst. Bars, 2 µm.

**Figure 6:**
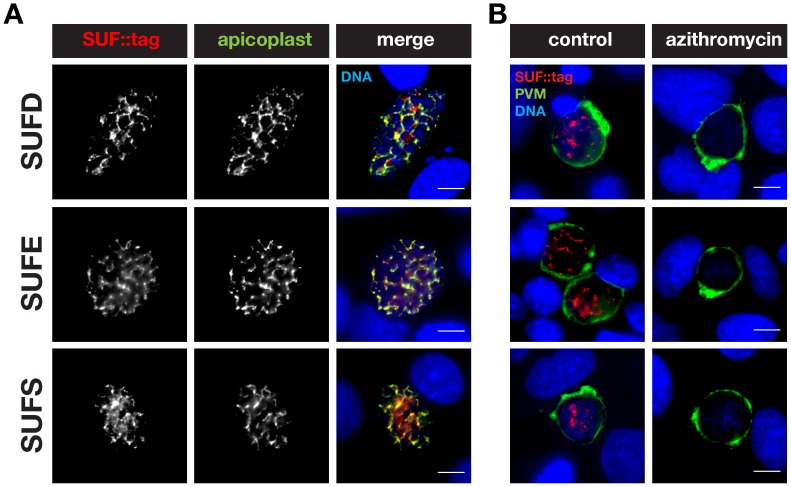
Localization of *Plasmodium berghei* SUFD, E, and S to the apicoplast in liver stages. (A) Co-staining of fixed, *sufD::tag*, *sufE::tag*, or *sufS::tag* parasite-infected hepatoma cells 48 h after sporozoite infection using anti-mCherry antibodies and anti-sera against acyl carrier protein (ACP). Note substantial overlap between the SUF::tag proteins and the signature apicoplast protein. (B) Drug treatment of *suf::tag*-infected hepatoma cells to corroborate apicoplast localization of the SUF::tag proteins. During liver stage development *suf::tag*-infected cells were left untreated (control) or treated with 1 µM azithromycin. Liver stages were stained with anti-mCherry antibodies and anti-sera against upregulated in infective sporozoite protein 4 (UIS4), a signature protein of the parasitophorous vacuolar membrane (PVM). Nuclei were stained with the DNA-dye Hoechst. Bars, 10 µm.

These parasites also provided an opportunity to verify the predicted apicoplast targeting by fluorescence microscopy (Fig. 5 and 6). As expected, all five fusion proteins displayed a punctate staining in live *P. berghei* blood stage trophozoites, reminiscent of the apicoplast (Fig. 5A). The localization of *Pb*SUFC::tag fully supports the previous finding of a punctate pattern in *P. falciparum*-infected erythrocytes observed with an anti-SUFC serum [27]. We confirmed the apparent apicoplast localization in fixed blood stage parasites by staining these with anti-mCherry antibodies and an anti-serum recognizing acyl carrier protein (ACP), an apicoplast signature protein (Fig. 5B). Using the same antibodies, we obtained supporting evidence for an apicoplast localization of three SUF fusion proteins, SUFD::tag, SUFE::tag, and SUFS::tag, in developing liver stage parasites (Fig. 6A). As expected in the case of an apicoplast localization, the characteristic, SUFD, E, and S-positive branched structures were destroyed following treatment with azithromycin (Fig. 6B).

We note that all tagged SUF proteins displayed similar localization, yet differed in intensity in the live blood stage parasites, with SUFC::tag yielding the most prominent signal, while SUFA::tag and SUFD::tag were only detectable after prolonged exposure times. Transcription data available online (http://PlasmoDB.org) confirm generally low transcript levels, particularly for *SUFA* and *SUFD*, with *SUFE* being most abundantly expressed [37]. Though we could not confirm this in live blood stage parasite, SUFE::tag signals were the most prominent during liver stage development. This initial observation indicates that a detailed biochemical study to investigate the stoichiometry and order of the apicoplast [Fe-S] cluster biogenesis pathway is warranted.

## Discussion

Our data provide the first genetics evidence that four of five SUF proteins are refractory to targeted gene deletion and, hence, most likely vital for blood stage development. We provide additional experimental support for this notion by successful fluorescent tagging through a complementation strategy. Based on the important role(s) of SUFC for parasite growth, we hypothesize that, by analogy, its partner protein SUFB, encoded in the apicoplast genome and inaccessible to experimental genetics, likely exerts essential functions as well.

Two aspects provide further confidence that the central components of the *Plasmodium* apicoplast SUF system, namely *SUFC*, *SUFD*, *SUFE*, and *SUFS*, are critical for survival during blood stage growth. First, we used the most recent and efficient techniques available for experimental genetics in *P. berghei* [34], [35] that have previously enabled the challenging generation of a slow growing recombinant line lacking a putative protein export regulator [38]. Second, we successfully deleted only two components of the apicoplast [Fe-S] cluster biogenesis pathway, namely *SUFA,* described herein, and *NFUapi* [32], both of which are estimated to have carrier rather than assembly functions in *E. coli* [19], [39]. This finding combined with the absence of a plastid-like SUF system in humans provides a rationale for further studies towards the development of antimalarial drugs targeting the *Plasmodium* SUF pathway.

[Fe-S] cluster biogenesis has been well studied in bacteria and yeast, and to a lesser extent in other eukaryotes [16], [24], [40]–[42]. However, very little functional data are yet available for apicomplexan parasites. The dispensable function of *Pb*SUFA could be compatible with a role as a transfer protein as suggested by studies in *E. coli* [19] rather than that of a scaffold protein. Similarly, we discussed a potential function of *Pb*NFUapi as a transfer protein [32]. Together, our data suggest that the putative transfer proteins in the apicoplast [Fe-S] biosynthesis pathway are not essential for parasite survival. Alternatively, both proteins might perform at least partially redundant functions, a possibility that might be further tested through the generation of recombinant parasite lines lacking both *NFUapi* and *SUFA*.

The corresponding SUF [Fe-S] cluster biogenesis pathway in the related apicomplexan parasite, *Toxoplasma gondii*, which causes toxoplasmosis, is likely also localized to the apicoplast, despite the uniform absence of a signal peptide and apicoplast-targeting sequences in the SUF proteins [24]. It remains elusive how this alternative targeting to the apicoplast might have evolved and if there are specific reasons why all of the SUF components in *T. gondii* are targeted through this alternative pathway. Apparently, other apicomplexan parasites, such as *Babesia bovis* and *Theileria annulata*, target these components through a classical signal peptide and apicoplast-targeting sequence-dependent import pathway, although these parasites appear to encode a reduced set of proteins, namely *SUFE* and *SUFS* and, in the case of *T. annulata*, also *NFUapi* [25], [32].

In *E. coli*, deficiencies in the SUF pathway do not result in phenotypes under normal growth conditions. However, when cultured in low iron or increased oxidative stress conditions, bacteria demonstrate marked growth problems [21], [43], [44]. As all our analyses were performed under optimized *in vivo* and cell culture conditions, we cannot exclude a phenotype of *sufA* ^–^ under suboptimal growth conditions, *e.g.* in malnourished mice.

*Plasmodium* is an obligate intracellular eukaryotic pathogen and apparently cannot compensate for the loss of the apicoplast SUF pathway. Recent data suggest that a functional non-mevalonate isoprenoid biosynthesis (DOXP) pathway is the major vital role of the apicoplast in *P. falciparum* parasites *in vitro* [31]. Presence of [Fe-S] clusters in the penultimate and ultimate enzymes, ISPG and ISPH, provide a plausible explanation for the observed refractoriness of the SUF assembly machinery to targeted gene deletion. To test if the DOXP pathway of isoprenoid biosynthesis is the sole reason for the essentiality of [Fe-S] cluster biogenesis in the apicoplast, one could attempt to delete *P. falciparum* SUF pathway components under isopentenyl pyrophosphate supplementation [31].

In conclusion, our study identified the four key components of the *Plasmodium* apicoplast [Fe-S] biosynthetic pathway and revealed that *SUFA* is dispensable for efficient progression through the *Plasmodium* life cycle.

## Materials and Methods

### Ethics Statement

This study was carried out in strict accordance with the German ‘Tierschutzgesetz in der Fassung vom 22. Juli 2009’ and the Directive 2010/63/EU of the European Parliament and Council ‘On the protection of animals used for scientific purposes’. The protocol was approved by the ethics committee of the Berlin state authority (‘Landesamt für Gesundheit und Soziales Berlin’, permit number G0469/09).

### Experimental Animals, Parasites, and Cell Lines

Female NMRI and C57BL/6 mice were purchased from Charles River Laboratories (Sulzfeld, Germany). C57BL/6 mice were used for sporozoite infections. All other parasite infections were conducted with NMRI mice. Experimental genetics were all performed in *P. berghei* strain ANKA (WT), as control lines GFPcon [33] or Berred [38] parasites were used. *In vitro* liver stage parasite development was analyzed using cultured HuH7 hepatoma cells.

### Generation of SUF Targeting and Tagging Plasmids

For targeted gene deletion of the *P. berghei SUF* genes, fragments of the upstream 5′ and downstream 3′ flanking regions (FR) were amplified from gDNA using gene-specific primers. PCR fragments were cloned into the *P. berghei* adaptable transfection vector (pBAT-SIL6) [34], which contains drug-selectable and high-expressing GFP cassettes. First, the 3′FR homologous sequences were cloned following restriction digestion of vector and insert with HindIII and KpnI. Then, the 5′FR homologous sequences digested with SacII and EcoRV were cloned into SacII and PvuII linearized vector, thus removing the mCherry-3xMyc tag from the original vector. The resulting plasmids were linearized with SalI. To provide transfection controls and confirm the apicoplast localization of SUF proteins, mCherry-3xMyc tagged parasite lines were generated. For this purpose, the carboxy-terminal parts of the *SUF* genes were PCR amplified using gene-specific primers. After restriction digestion, the respective fragments were cloned into the SacII and HpaI digested pBAT-SIL6 vector already containing the 3′FR sequence of the respective *SUF* genes, thus fusing the *SUF* carboxy-terminal sequence in frame with the mCherry-3xMyc tag sequence. The resulting plasmids were linearized with SalI. All primers are listed in Table S1.

### Parasite Transfection, Selection and Genotyping of Recombinant Parasites

For targeted gene deletions and carboxy-terminal tagging, 10^6^ to 10^7^ purified *P. berghei* schizonts were transfected with digested plasmids using the Amaxa Nucleofector system as described [33]. Transfected parasites were subsequently injected into naïve NMRI mice selected by oral pyrimethamine (70 µg/ml) in the drinking water. Genotyping of drug-resistant parasites was performed by diagnostic PCR using gDNA as template and integration-specific primers. Two isogenic *sufA* ^–^ parasite lines from two independent transfection experiments were generated by flow cytometry-assisted isolation as described [35]. The genotype of the two selected *sufA^–^* parasite populations was confirmed by Southern blot analysis using the PCR DIG Probe Synthesis kit and the DIG Luminescent Detection kit (Roche), according to the manufacturer’s protocol. For amplification of the hybridization probe, gene-specific primers TV-5’SUFA-F and TV-5’SUFA-R were used. The hybridization probe was annealed to EcoRI restriction-digested gDNA, resulting in bands of 4.5 kb (WT) and 9.8 kb (*sufA* ^–^). All primers are listed in Table S1.

### *Plasmodium* Life Cycle Progression

Gametocyte differentiation and exflagellation of microgametes were examined prior to mosquito feeding. *Anopheles stephensi* mosquitoes were raised under a 14 h light/10 h dark cycle, 75% humidity and at 28°C (non-infected) or 20°C (infected), respectively. Sporozoite populations were isolated and analyzed as described previously [45]. Mosquito infectivity, *i.e.* the percentage of mosquitoes with midgut oocysts, was assessed through dissection of mosquito midguts at day 10 after feeding. All parasite strains that were used express GFP in all life cycle stages, allowing the determination of the number of midguts containing GFP-positive oocysts using a fluorescence binocular. Salivary gland-associated sporozoites were quantified at days 17–21. To determine sporozoite infectivity, sporozoites were liberated from salivary glands and injected intravenously into young, naïve C57BL/6 mice (10,000 sporozoites/inoculation). Patency was determined by daily examination of Giemsa-stained thin blood smears.

*P. berghei in vitro* liver stages were cultured and analyzed using standard techniques [46]. In brief, 30,000 hepatoma cells were seeded per well in 8-well chamber slides (Nalge Nunc International) and inoculated with freshly-dissected 10,000 sporozoites 24 h later. Thereafter, standard procedures for culturing infected hepatoma cells were followed [47]. Merosomes were harvested from the cell culture supernatant and counted in a Neubauer chamber 72 h after inoculation.

### Fluorescence Microscopy

For confirmation of expression and determination of the subcellular localization of tagged SUF proteins, live and fixed *suf::tag* blood stage parasites were imaged using Leica DMR epifluorescence microscope. Infected erythrocytes were fixed using a previously published protocol with minor modifications [48]. 5 µl of tail blood from an infected mouse was mixed with 125 µl of RPMI1640 and 15 µl of cell suspension was allowed to settle 5 min onto poly-L-lysine coated cover slips. Cover slips were transferred to a 24-well plate containing 500 µl of 4% EM-grade paraformaldehyde and 0.0075% EM-grade glutaraldehyde in microtubule stabilizing buffer (MTSB, 10 mM MES, 150 mM NaCl, 5 mM EGTA, 5 mM glucose, 5 mM MgCl_2_ pH 6.9), fixed for 20 min and washed with PBS. Cells were permeabilized for 10 min with 0.1% Triton X-100 in PBS and blocked 3 h with 10% foetal calf serum (FCS) in PBS. The samples were incubated with rat anti-mCherry antibodies (1∶1,000 dilution, Chromotek) and rabbit anti-*P. berghei* ACP peptide antiserum (1∶300 dilution; [49]) in 10% FCS in PBS overnight at 4°C. Bound antibodies were detected using goat anti-rat IgG Alexa Fluor 546 and goat anti-rabbit IgG Alexa Fluor 633 conjugated antibodies (1∶3,000 dilution, Invitrogen). Nuclei were visualized with the DNA-dye Hoechst 33342 (Invitrogen; 1∶1,000 dilution). Coverslips were mounted with Fluoromount-G (Southern Biotech).

Liver stages were fixed with ice-cold methanol for immunofluorescence assays at the indicated time points. For quantification of *sufA* ^–^ liver stage parasite numbers, these were visualized using monoclonal mouse anti-*P. berghei* heat shock protein 70 (HSP70) antibodies (1∶300 dilution) [50]. For confirmation of expression and determination of the subcellular localization of the SUF::tag proteins 48 h after infection, fixed *suf::tag* liver stage parasites were incubated with rat anti-mCherry antibodies (1∶1,000 dilution, Chromotek) and rabbit anti-*P. berghei* ACP peptide antiserum (1∶300 dilution; [49]). To confirm the apicoplast localization at 48 h after infection, we treated sporozoite-infected hepatoma cells with 1 µM azithromycin (Pfizer), as described previously [46]. Parasites were identified by staining with rabbit anti-upregulated in infective sporozoites protein 4 (UIS4) peptide antiserum (1∶2,000 dilution; kindly provided by G. Montagna, MPI-IB, Berlin). Branched anti-mCherry-positive structures extending into the area delineated by the anti-UIS4 antiserum were defined as apicoplasts. Bound antibodies were detected using donkey/goat anti-rabbit/rat/mouse IgG Alexa Fluor 488/546 conjugated antibodies (1∶3,000 dilution, Invitrogen). Nuclei were visualized with DNA-dyes Hoechst 33342 (Invitrogen) and DRAQ5 (Axxora; both 1∶1,000 dilution). Coverslips were mounted with Fluoromount-G (Southern Biotech). Total numbers of parasites were counted using a Leica DM2500 epifluorescence microscope. Images were recorded using a Zeiss AxioObserver Z1 epifluorescence microscope.

All images were processed minimally with ImageJ (http://rsb.info.nih.gov/ij/). Following subtraction of background fluorescence levels of non-infected cells within the same recording, minimum and maximum intensities of the specific signals were optimized to use the full dynamic range of the look-up-tables. No gamma adjustments were applied.

## Supporting Information

Table S1 **Primer sequences.**
(DOCX)
