# Supplementary material for: Identification of Vital and Dispensable Sulfur Utilization Factors in the Plasmodium Apicoplast
Source: PLoS One. 2014 Feb 21;9(2):e89718. doi: 10.1371/journal.pone.0089718 (PMC3931816; doi:10.1371/journal.pone.0089718)
Supplement: Table S1 — Primer sequences. (DOCX) [file pone.0089718.s001.docx]

| **Table S1.** Primer sequences. | | | | |
| --- | --- | --- | --- | --- |
| **Primer Name^a^** | **Primer Sequence (REase sites underlined)** | **REase** | **Size WT (bp)^b^** | **Size INT (bp)^c^** |
| **Primers of general use** | | | | |
| 5’HSP70rev^d^ | CAATTTGTTGTACATAAAATAGGCAG |  |  |  |
| 5’DHFRrev^d^ | ATGAAATACCGCTCCATTTTTCC |  |  |  |
| ***SUFA* - PBANKA_123740** | | | | |
| TV-5’SUFA-F | TTTCCGCGGTTTTAGTTTATTATATTTCACAATTTGCAC | SacII | 503 |  |
| TV-5’SUFA-R | AAAGATATCATAATGAGGGGTAAATGAACCAG | EcoRV |  |  |
| TV-C-SUFA-F | TATCCGCGGTGTAGAAAATGGGGGTTGTAAAGG | SacII | 536 |  |
| TV-C-SUFA-R | ATTGATATCGACATTAAAGGATTTTCCACAACCAC | EcoRV |  |  |
| TV-3’SUFA-F | TTTAAGCTTTTGTATGGTCCCCTATATGAATTTG | HindIII | 466 |  |
| TV-3’SUFA-R | TTTGGTACCGATGCATTTTTGTTAAGTTTGTGC | KpnI |  |  |
| GT-5’SUFA-F | TTCATCTCCTTTTGGCTATTTTG |  | 819 | 812 |
| GT-5’SUFA-R | TAAAGCCAGCGTATCTCAAG |  |  |  |
| GT-C-SUFA-F | CACCAACTACATGCGTATGTG |  | 788 | 2362 |
| GT-C-SUFA-R | TTTGATTAACGATCGAAATATAACACC |  |  |  |
| GT-3’SUFA-F | GAAAAAATGTGGTTGTGGAAAATCC |  | 634 |  |
| GT-3’SUFA-R | AAAATGGAGAAAACAGGGTTACG |  |  | 1229 |
| ***SUFC* - PBANKA_102920** | | | | |
| TV-5’SUFC-F | TTTCCGCGGATGCTTATCCATTTTGCTTGG | SacII | 527 |  |
| TV-5’SUFC-R | TAAGATATCGTGTCACATGTTGTATTTCCTTTC | EcoRV |  |  |
| TV-C-SUFC-F | AAACCGCGGCAGGAAAATCAACACTTGCTAAAG | SacII | 629 |  |
| TV-C-SUFC-R | AAAGATATCTTCCTCAACAAATTGTGCATATCC | EcoRV |  |  |
| TV-3’SUFC-F | ATTAAGCTTATTTATTGCATGCCCTTGTTTTG | HindIII | 616 |  |
| TV-3’SUFC-R | TTTGGTACCTGCTTACACAATTATCTCTTTTTGG | KpnI |  |  |
| GT-5’SUFC-F | TTGCTATCTATTGTTATCATATTTCTTG |  | 631 | 721 |
| GT-5’SUFC-R | CTAGCCAAAATTGTCCTCGC |  |  |  |
| GT-C-SUFC-F | ACAATCACCTGCTTGGAATG |  | 1392 | 2258 |
| GT-C-SUFC-R | = TV-3’SUFC-R |  |  |  |
| GT-3’SUFC-F | AATCTGATGGATATGCACAATTTG |  | 929 |  |
| GT-3’SUFC-R | AAAATCATCGCCATATTCTATATTACC |  |  | 1519 |
| ***SUFD* - PBANKA_094350** | | | | |
| TV-5’SUFD-F | TATCCGCGGCATATTTTTTGTTCTTTTTCCTATTCACC | SacII | 491 |  |
| TV-5’SUFD-R | AAAGATATCCATTAAAGCTATCCAAAAGAAAGTG | EcoRV |  |  |
| TV-C-SUFD-F | TTTCCGCGGTTTGGGGAGTTTATCATCACGTG | SacII | 562 |  |
| TV-C-SUFD-R | TTTGATATCTAGATTAGTCATACGTAATATTTTTTTTGATTC | EcoRV |  |  |
| TV-3’SUFD-F | TTTAAGCTTTATTCCATGCAGCACATTGG | HindIII | 523 |  |
| TV-3’SUFD-R | TTTGGTACCGCCTGTGAAGCTCAATTTCG | KpnI |  |  |
| GT-5’SUFD-F | TCGAACCCATGCGAACTTAC |  | 888 | 850 |
| GT-5’SUFD-R | TTCGTTGCTTCTTTTCTTTTCAC |  |  |  |
| GT-C-SUFD-F | ACAAACCCGCGATTAGTCC |  | 1330 | 2281 |
| GT-C-SUFD-R | = TV-3’SUFD-R |  |  |  |
| GT-3’SUFD-F | TTCTTGAACATGTGCCTGATG |  | 709 |  |
| GT-3’SUFD-R | AGGACCATGCAAAGGACCTC |  |  | 1210 |

| **Table S1.** Primer sequences (continued). | | | | |
| --- | --- | --- | --- | --- |
| **Primer Name^a^** | **Primer Sequence (REase sites underlined)** | **REase** | **Size WT (bp)^b^** | **Size INT (bp)^c^** |
| ***SUFE* - PBANKA_030380** | | | | |
| TV-5’SUFE-F | TTTCCGCGGTGTGCCATTATCGCATTACAG | SacII | 578 |  |
| TV-5’SUFE-R | AAAGATATCAAAAGGCCTACATAACATTCCAG | EcoRV |  |  |
| TV-C-SUFE-F | AAACCGCGGAATGAGTTGAAAACAAGGCAAAATC | SacII | 519 |  |
| TV-C-SUFE-R | TTTGATATCGTGTTCTATATTTTTAATAATTTGCTCG | EcoRV |  |  |
| TV-3’SUFE-F | TTTAAGCTTGAATACAATGCATGCTATATAATGC | HindIII | 599 |  |
| TV-3’SUFE-R | TATGGTACCTTTTAATGCAAAAATGCACAAGG | KpnI |  |  |
| GT-5’SUFE-F | CTGGCGTTTTGTGTCCATTAC |  | 994 | 905 |
| GT-5’SUFE-R | TAATTTTGTGCGAATGATCGAC |  |  |  |
| GT-C-SUFE-F | GCTTATCGGGGAGTCAATTTC |  | 1560 | 2570 |
| GT-C-SUFE-R | = TV-3’SUFE-R |  |  |  |
| GT-3’SUFE-F | CTAATTTGTCCTTGTTTCGTTGTC |  | 1104 |  |
| GT-3’SUFE-R | TGTAACAATAAAATAAGACGGTTGG |  |  | 1646 |
| ***SUFS* - PBANKA_061430** | | | | |
| TV-5’SUFS-F | TTTCCGCGGGATTTTGCCTTTTTCAAGAATATGG | SacII | 571 |  |
| TV-5’SUFS-R | ATAGATATCCTCATTATTCATATTCCCACAAATTTG | EcoRV |  |  |
| TV-C-SUFS-F | ATACCGCGGCTACATCTTTGAAACCGGAACTC | SacII | 601 |  |
| TV-C-SUFS-R | TTAGTTAACAACAGCTTTTCCATTTCTACGC | HpaI |  |  |
| TV-3’SUFS-F | AAAAAGCTTATCATTTGTGTGTGCTCATACG | HindIII | 574 |  |
| TV-3’SUFS-R | AAAGGTACCATTCTCCATGCAAAGCAAAAATAC | KpnI |  |  |
| GT-5’SUFS-F | CATAAGCGAGCCACACATTG |  | 764 | 817 |
| GT-5’SUFS-R | TATATGTCCTTTATAAATTGTACGTGTG |  |  |  |
| GT-C-SUFS-F | TGTGCTCCATTTGGTAGTGG |  | 1363 | 2215 |
| GT-C-SUFS-R | = TV-3’SUFS-R |  |  |  |
| GT-3’SUFS-F | TCAGGACACCACTGTGCATC |  | 943 |  |
| GT-3’SUFS-R | TTGTTTGATTTTCACACGCTTTG |  |  | 1338 |

^a^ TV, primers used for construction of Transfection Vectors; GT, primers used for GenoTyping.

^b^ Sizes of the PCR products of forward and reverse primers on WT gDNA.

^c^ Sizes of the respective integration-specific PCR products; forward 5’ gene-specific primers combined with 5’HSP70rev and reverse 3’ gene-specific primers combined with 5’DHFRrev.

^d^ Reference: Kenthirapalan S, Waters AP, Matuschewski K, Kooij TWA (2012) Flow cytometry-assisted rapid isolation of recombinant *Plasmodium berghei* parasites exemplified by functional analysis of aquaglyceroporin. Int J Parasitol 42: 1185-1192.
